# Patient Satisfaction in Malaysia's Busiest Outpatient Medical Care

**DOI:** 10.1155/2015/714754

**Published:** 2015-01-12

**Authors:** Kurubaran Ganasegeran, Wilson Perianayagam, Rizal Abdul Manaf, Saad Ahmed Ali Jadoo, Sami Abdo Radman Al-Dubai

**Affiliations:** ^1^Medical Department, Tengku Ampuan Rahimah Hospital (HTAR), Jalan Langat, 41200 Klang, Selangor, Malaysia; ^2^Department of Community Health, UKM Medical Centre, Jalan Yaacob Latif, Cheras, 56000 Kuala Lumpur, Malaysia; ^3^International Institute for Global Health, United Nations University, Jalan Yaacob Latif, Cheras, 56000 Kuala Lumpur, Malaysia; ^4^International Centre for Case-Mix and Clinical Coding, UKM Medical Centre, Jalan Yaacob Latif, Cheras, 56000 Kuala Lumpur, Malaysia; ^5^Department of Community Medicine, International Medical University, Jalan Jalil Perkasa, Bukit Jalil, 57000 Kuala Lumpur, Malaysia

## Abstract

This study aimed to explore factors associated with patient satisfaction of outpatient medical care in Malaysia. A cross-sectional exit survey was conducted among 340 outpatients aged between 13 and 80 years after successful clinical consultations and treatment acquirements using convenience sampling at the outpatient medical care of Tengku Ampuan Rahimah Hospital (HTAR), Malaysia, being the country's busiest medical outpatient facility. A survey that consisted of sociodemography, socioeconomic, and health characteristics and the validated Short-Form Patient Satisfaction Questionnaire (PSQ-18) scale were used. Patient satisfaction was the highest in terms of service factors or tangible priorities, particularly “technical quality” and “accessibility and convenience,” but satisfaction was low in terms of service orientation of doctors, particularly the “time spent with doctor,” “interpersonal manners,” and “communication” during consultations. Gender, income level, and purpose of visit to the clinic were important correlates of patient satisfaction. Effort to improve service orientation among doctors through periodical professional development programs at hospital and national level is essential to boost the country's health service satisfaction.

## 1. Introduction

Patient satisfaction is defined as a subjective evaluation of the health service received against client's expectations [1]. It is principally evaluated over seven health service dimensions: general satisfaction, technical quality, interpersonal aspects, communication, financial aspects, time spent with doctor, and the ease of contact or availability [2]. Perceived positive or negative reactions by patients to salient features of the context, process, or procedures and outcomes of the healthcare client charter are vital to monitor the quality of care provided in a health facility [3]. Patient satisfaction prompts compliance with medical advice and treatment, service utilization, and enhancement of the doctor-patient relationship [4].

The Malaysian public healthcare sector is plagued by the influx of patient admissions, propagating surplus demand of uneven population-healthcare provider ratio and perceptions of poor service quality [5]. In addition to long waiting times, emotional burnout, service orientation of doctors, particularly doctor's professionalism, the lack of empathy, poor level of competencies, aggressive pursuit of monetary gains, and their disregard for patient suffering in medical practice had caused substantial dissatisfaction towards public healthcare service providers [4, 6, 7]. This study aimed to explore the factors affecting patient satisfaction with outpatient medical care in Malaysia.

## 2. Methods

### 2.1. Ethics Statement

This study complied with the guidelines convened in the Declaration of Helsinki. Study protocol was approved by the Medical Research Ethics Committee (MREC), Ministry of Health Malaysia (government approval number: NMRR-13-643-14711). Objectives and benefits of the study were explained in verbal and written form attached to the questionnaires in a sealed envelope. Patients were assured that their participation was confidential and would not affect their medical treatment outcomes. Upon completion of the questionnaire, patients were instructed to put those sealed envelopes into a collection-box placed at the clinic's counter. A written consent was obtained from those who agreed to participate. In addition, a written informed consent from the next of kin, caretakers, or guardians on behalf of the minors aged 13–19 years was obtained in this study.

### 2.2. Design, Setting, and Participants

This cross-sectional study was conducted for two weeks in the month of December 2013 at the outpatient medical care of Tengku Ampuan Rahimah Hospital (HTAR), Malaysia, the country's busiest medical outpatient facility [8]. Trained data collectors with the assistance of a clinic nurse approached patients aged between 13 and 80 years after successful clinical consultations and treatment acquirements using convenience sampling. The total sample size was calculated to be 384 based on 50% expected prevalence rate of health service satisfaction [1] and 0.05 level of accuracy [1, 9]. Forty-four questionnaires were excluded due to missing data. The total response rate was 88.5% (340/384).

The completed self-administered questionnaires were recollected back by the trained data collectors in a sealed envelope from the collection-box placed in the clinic. Patients acquiring treatment for acute illness, illiterate patients who were unable to converse or interpret the study questionnaire, and those refusing participation were excluded from the study.

### 2.3. Study Instruments

A self-administered questionnaire consisting of three parts was used in this study.

The first part included items on sociodemographic factors (gender, age, and marital status).

The second part included socioeconomic and health characteristics of the patients (household income, employment status, education level, type of residence, overall health perception, and purpose of visit to clinic).

To explore patient satisfaction towards healthcare service, we used the Short-Form Patient Satisfaction Questionnaire (PSQ-18), originally developed by Marshall and Hays [10] and validated among Malaysian population [3, 11]. This widely used self-reported tool assessed health service satisfaction in various clinical settings, including primary healthcare clinics and hospital based outpatient departments [3, 11, 12]. PSQ-18 is comprised of eighteen items with seven dimensions which measures general satisfaction (2 items), technical quality (4 items), interpersonal manner (2 items), communication (2 items), financial aspects (2 items), time spent with doctor (2 items), and accessibility and convenience (4 items). These items were scored on a five-point Likert scale ranging from one (strongly agree) to five (strongly disagree). Each dimension that aimed to explore tangible priorities and patient experience on health service satisfaction is evaluated through different related questions that identify a particular area for improvement. Some PSQ-18 items are worded so that agreement reflects satisfaction with medical care, whereas other items were worded so that agreement reflects dissatisfaction with medical care. We reversed the scores of items worded that reflected disagreement with medical care to tabulate the total satisfaction score [10]. A higher score reflected greater satisfaction with medical care. After item scoring, items within the same subscale were averaged together to create the seven subscaled scores [10].

### 2.4. Statistical Analysis

Analysis was performed using Statistical Package for Social Sciences (SPSS) (version 16.0, IBM, Armonk, NY). Descriptive analysis was performed for all variables in this study. To check for the factor structure of the PSQ-18 among Malaysian outpatients, an exploratory factor analysis was performed using principal component analysis with direct oblimin method. Cronbach's alpha was used to test the internal consistency of the scale. Patient satisfaction scores were expressed as mean and standard deviations. Test of normality was performed for total satisfaction scores. The *t*-test and ANOVA test were applied to compare the total score of patient satisfaction across sociodemographic, socioeconomic, and health characteristic variables. In case of ANOVA, post hoc test was used to determine where the significant difference was. Multiple linear regression analysis using “Enter” technique was employed to obtain the significant factors associated with patient satisfaction scores. The accepted level of significance was set below 0.05 (*P* < 0.05). Multicollinearity was checked between independent variables.

## 3. Results

### 3.1. Sample Characteristics

A total of three hundred forty patients (88.5% response rate) consented to participate in this study. Of the total, 211 (62.1%) were males and 129 (37.9%) were females. The mean (±SD) age of the patients was 40.7 (±19.4) years and the majority aged less than 30 years 141 (41.5%). The majority of the patients were married 230 (67.6%). Most of the patients had tertiary education 201 (59.1%) and were employed 219 (64.4%) with a monthly household income of MYR 3000 or more 261 (76.8%). The majority of the patients were residing in an urban area 263 (77.4%). Most patients perceived a “good” health status 275 (80.8%), and the majority came to the clinic for “follow-up treatment” 270 (79.4%) (Table 1).

### 3.2. Patient Satisfaction with Outpatient Medical Care

Table 2 exhibits patient satisfaction score with outpatient medical care. Cronbach's alpha values for the seven subscales of PSQ-18 had values ranging between 0.63 and 0.79. The mean (±SD) of patient satisfaction score was the highest in terms of institutional factors, particularly “technical quality” (14.2 ± 1.8), followed by “accessibility and convenience” to healthcare service (10.9 ± 2.4). The mean (±SD) of patient satisfaction score was the lowest in terms of service orientation, particularly “time spent with doctor” during consultations (5.7 ± 1.3). The mean (±SD) of general satisfaction towards healthcare service acquired by patients scored average (7.7 ± 1.2). The mean (±SD) of the total satisfaction score was 59.2 ± 6.5 and the score ranged between 34 and 87.

### 3.3. Association between Sample Characteristics and Outpatient Medical Care Satisfaction

Male patients perceived a significantly higher service satisfaction (60.0 ± 6.9) compared to females (58.0 ± 5.7) (*P* = 0.005). There was a significant association between age and service satisfaction (*P* = 0.001); a post hoc test revealed that patients aged 50 years or more perceived higher service satisfaction (61.4 ± 8.1) in comparison to patients aged 30–49 years (59.0 ± 7.5, *P* = 0.033) and those aged less than 30 years (58.1 ± 3.7, *P* = 0.001). Patients with a monthly household income of less than MYR 3000 perceived higher service satisfaction (63.4 ± 10.2) when compared to those with higher income (58.0 ± 4.2) (*P* < 0.001). Patients with high school education exhibited higher service satisfaction (60.4 ± 8.1) than tertiary graduates (58.4 ± 5.1) (*P* = 0.004). There was a significant association between purpose of visit to the clinic and the health service satisfaction (*P* < 0.001); a post hoc test revealed that newly referred patients perceived higher service satisfaction (61.0 ± 8.4) in comparison to patients acquiring follow-up treatment (59.1 ± 5.7, *P* = 0.002) or other medical services (51.6 ± 10.0, *P* < 0.001) (Table 3).

### 3.4. Factors Associated with Outpatient Medical Care Satisfaction among Patients by Multiple Linear Regression

Male patients had on the average 1.4 (95% CI 0.1–2.7) higher service satisfaction score as compared to females (*P* = 0.037). Patients with a monthly household income of less than MYR 3000 had on the average 5.0 (95% CI 3.4–6.5) higher service satisfaction score compared to those with higher income (*P* < 0.001). Patients acquiring “follow-up treatment” had on the average 7.6 (95% CI 3.7–11.6) higher service satisfaction score than patients acquiring other medical services (*P* < 0.001). Newly referred patients had on the average 9.2 (95% CI 5.0–13.3) higher service satisfaction score in comparison to patients acquiring other medical services (*P* < 0.001) (Table 4).

## 4. Discussion

This study aimed to explore the factors affecting patient satisfaction with outpatient medical care in Malaysia. Cronbach's alpha values of the PSQ-18 subscales had values greater than 0.70 suggesting an acceptable internal consistency except for two subscales (interpersonal manner and communication scales) that fall below the lower limit. These values were consistent with the original psychometric properties concluded by Marshall and Hays [10]. In the final model, gender, income level, and purpose of visit to the clinic exhibited significant associations with patient satisfaction scores.

Newly referred cases exhibited the highest service satisfaction, followed by patients returning for a clinical review. The implementation of “*e-masa*” policy into the Malaysian healthcare service delivery system at all hospital based outpatient departments or primary healthcare clinics nationwide that aimed to provide patient contact within 30 minutes of newly registered cases by a healthcare provider would support the prevailing association in this study [8]. Healthcare service satisfaction would predict patient's intention to return for similar services in the future [1, 13]. In this study, follow-up cases showed significant association with patient satisfaction scores, consistent with previous findings [1, 14]. Patient's experience to a previously satisfied health service encounter would prevent navigation to different healthcare facilities [1, 10].

This study found a significantly higher service satisfaction among patients with a lower income in comparison to those with higher income. Equity and accessibility to healthcare services have always been the commitment given by the Malaysian government, with the public healthcare sector subsidizing nearly 95% of the patients' cost for treatment, allowing healthcare access to over 90% of the total population [5]. With a minimal registration fee of USD 0.33 or MYR 1, Malaysians are granted free access to clinical consultations, treatment, and medications as both outpatients and inpatients in all public health facilities within the country [8].

This study found that patients with high school education perceived a significantly higher service satisfaction in comparison to tertiary graduates, inconsistent with a previous finding from Uganda [1]. The lower patient satisfaction score relative to high school education may be due to a potentially greater service expectation by the more highly educated patients. Sociodemographic characteristics of patients have been reported to be associated with patient's health service satisfaction [15]. This study found significant associations between gender, age, and patient's health service satisfaction.

Health service ratings concerning “technical quality,” “accessibility and convenience,” and doctors “interpersonal skills” are good predictors for patient satisfaction [16]. This study found that patient ratings on “technical quality” and “accessibility and convenience” had the highest satisfaction score but ratings on “interpersonal skills” satisfaction declined. Similar consistencies with different magnitude of satisfaction scores between these two dimensions were observed in a previous study [16]. Professionalism, particularly communicative attitudes, and empathy for patient suffering are vital to enhance the doctor-patient relationship [4], which improves patient satisfaction [11].

This study found that patient satisfaction score was the lowest in terms of service orientation of doctors. Previous studies from developing countries found similar consistencies [6, 17]. The experienced and reputed doctors opting for the more lucrative private sector had resulted in shortages in the public health service sector. The existing doctors that remained to serve the public healthcare sector were incapable of giving due time and attention to patients during consultations, causing reduced patient satisfaction. This imbalance of resource distribution and workload had caused inequities, resulting in long queues for outpatient services, diagnostic procedures, treatment modalities, and rationing of drugs for chronic diseases within the public health service sector [5].

The mean general patient satisfaction scored average, suggesting the need for improvement. Our study was conducted in a general public hospital setting, where patients seek acute and chronic care with high expectations of quality health services compared to what could unrealistically be met, resulting in an average general satisfaction. Studies conducted in government teaching hospitals [11], government linked district, and state level hospitals [18] showed mixed variations of patient satisfaction score. These inconsistencies could be due to differences in healthcare services provided across different geographical settings among patients with different expectations and experiences [1].

## 5. Limitations

The cross-sectional nature of the study limits our ability to find causal inferences. Self-reported data collected at one point in time necessitated care in drawing conclusions owing to effects that staffs of the clinic would have been aware of; the study might affect the health service provided. Data obtained in this study was from a single hospital in Malaysia; this may affect the generalizability of the results to all medical outpatients in Malaysia. The sample size calculated for this study could not be achieved and may affect the power of the study. A larger and representative sample is recommended in future studies to avoid possible selection bias.

## 6. Conclusion

Patient satisfaction was the highest in terms of service factors or tangible priorities, particularly “technical quality” and “accessibility and convenience,” but satisfaction was low in terms of service orientation of doctors, particularly the “time spent with doctor,” “interpersonal manners,” and “communication” during consultations. Gender, income level, and purpose of visit to the clinic were important correlates of patient satisfaction. Periodical professional development training programs for doctors at hospital and national level under supervision by the Ministry of Health Malaysia to increase interpersonal competencies and communication skills are recommended to boost medical professionalism.

## Figures and Tables

**Table 1:** Sample characteristics (*n* = 340).

Characteristics	*N*	Percentage (%)
Gender		
Male	211	62.1
Female	129	37.9
Age (years)		
<30	141	41.5
30–49	111	32.6
≥50	88	25.9
Marital status		
Single	110	32.4
Married	230	67.6
Monthly household income (MYR)		
<3000	79	23.2
≥3000	261	76.8
Current employment status		
Employed	219	64.4
Unemployed	15	4.4
Student	66	19.4
Retired	40	11.8
Highest education level		
High school	139	40.9
Tertiary education	201	59.1
Residence		
Urban	263	77.4
Rural	77	22.6
Overall health perception		
Very good	7	2.1
Good	275	80.8
Fair	53	15.6
Bad	5	1.5
Very bad	0	0
Purpose of visit to clinic		
Follow-up treatment	270	79.4
Newly referred case	61	18.0
Others	9	2.6

^*^1 MYR is equivalent to 0.33 USD at the time of study.

**Table 2:** Subscale reliability of PSQ-18 and health service satisfaction scores (*n* = 340).

Characteristics	Cronbach's alpha	Scores mean (±SD)
*General satisfaction *	0.75	7.7 (1.2)
The medical care I have been receiving is just about perfect.		
I am dissatisfied with some things about the medical care I receive.		
*Technical quality *	0.74	14.2 (1.8)
I think my doctor's office has everything needed to provide complete medical care.		
Sometimes doctors make me wonder if their diagnosis is correct.		
When I go for medical care, they are careful to check everything when treating and examining me.		
I have some doubts about the ability of the doctors who treat me.		
*Interpersonal manner *	0.65	6.9 (1.3)
Doctors act like forced to treat or too impersonal towards me.		
My doctors treat me in a very friendly and courteous manner.		
*Communication *	0.63	7.5 (1.1)
Doctors are good about explaining the reason for medical tests.		
Doctors sometimes ignore what I tell them.		
*Financial aspects *	0.74	6.4 (1.2)
I feel confident that I can get the medical care I need without being set back financially.		
I have to pay for more of my medical care than I can afford.		
*Time spent with doctor *	0.79	5.7 (1.3)
Those who provide my medical care sometimes hurry too much when they treat me.		
Doctors usually spend plenty of time with me.		
*Accessibility and convenience *	0.75	10.9 (2.4)
I have easy access to the medical specialists I need.		
Where I get medical care, people have to wait too long for emergency treatment.		
I find it hard to get an appointment for medical right away.		
I am able to get medical care whenever I need it.		

**Table 3:** Association between sample characteristics and health service satisfaction (*n* = 340).

Characteristics	Mean (SD)	*P* value
Gender		0.005
Male	60.0 (6.9)	
Female	58.0 (5.7)	
Age (years)		0.001
<30	58.1 (3.7)	
30–49	59.0 (7.5)	
≥50	61.4 (8.1)	
Marital status		0.288
Single	58.7 (5.1)	
Married	59.5 (7.1)	
Monthly household income (MYR)		<0.001
<3000	63.4 (10.2)	
≥3000	58.0 (4.2)	
Current employment status		0.267
Employed	59.4 (7.1)	
Unemployed	59.0 (6.1)	
Student	59.9 (6.2)	
Retired	57.4 (2.9)	
Highest education level		0.004
High school	60.4 (8.1)	
Tertiary education	58.4 (5.1)	
Residence		0.078
Urban	58.9 (6.5)	
Rural	60.4 (6.4)	
Overall health perception		0.347
Very good	55.1 (9.8)	
Good	59.4 (6.4)	
Fair	58.8 (6.8)	
Bad	58.2 (2.6)	
Purpose of visit to clinic		<0.001
Follow-up treatment	59.1 (5.7)	
Newly referred case	61.0 (8.4)	
Others	51.6 (10.0)	

**Table 4:** Factors associated with health service satisfaction among patients by multiple linear regression (*n* = 340).

Predictors	*B*	SE	Beta	*P* value	95% CI lower–upper
Gender (male)	1.4	0.7	0.1	0.037	0.1–2.7
Age (≥50 years)	1.5	0.8	0.1	0.055	−0.1–3.0
Income (<MYR 3000)	5.0	0.8	0.3	<0.001	3.4–6.5
Follow-up treatment	7.6	2.0	0.5	<0.001	3.7–11.6
Newly referred case	9.2	2.1	0.5	<0.001	5.0–13.3

The reference group for gender is “female,” for age is “less than 30 years,” for income level is “≥MYR 3000,” and for purpose of visit is “others.”
